# Electronic communication in autism spectrum conditions

**DOI:** 10.1186/s13229-020-00329-2

**Published:** 2020-06-05

**Authors:** Lucy Anne Livingston, Chris Ashwin, Punit Shah

**Affiliations:** 1 School of Psychology, Cardiff University, Cardiff, UK; 2 Social, Genetic and Developmental Psychiatry Centre, Institute of Psychiatry, Psychology and Neuroscience, King’s College London, London, UK; 3 National Autistic Society, London, UK; 4 Department of Psychology, University of Bath, Bath, UK; 5 Centre for Applied Autism Research, Department of Psychology, University of Bath, Bath, UK

**Keywords:** Autism, Social communication, E-mail, Diagnosis, Human computer interaction

A core diagnostic feature of autism spectrum conditions (ASC) is atypical social communication. Considerable research, spanning multiple levels of explanation, has sought to establish the mechanisms underlying atypical social behaviour to aid diagnosis of ASC [1]. Nonetheless, ASC is still measured at the behavioural level, typically using semi-structured instruments during in-person and telephone interviews [2]. However, these procedures, though widely used in research and clinical settings, do not capture naturalistic social communication *before* an autistic adult visits the clinic/lab or outside of this environment. More ecologically valid, real-world measurements of autistic social communication are therefore required. We suggest that one such underexplored, yet potentially important, naturalistic opportunity to measure social-communicative behaviour—towards better understanding and support for autistic people—is electronic communication.

When scheduling research assessments, we consistently notice an atypical social-communicative style in e-mails from adults with ASC compared to non-autistic participants. Like most researchers, we had considered e-mail communication simply as the means to an end for organising neuropsychological testing. However, we now suggest that these naturalistic and socially relevant electronic interactions may be informative for investigating communication in ASC. Importantly, our e-mails sent to autistic and non-autistic adults (e.g., inviting them to participate in a study) are identical; therefore, the groups are broadly age-, gender- and IQ-matched for the lab-based studies in which they were invited to participate. In addition, our e-mails were neither designed nor structured to elicit a response for formal analysis. Together, this has created a controlled, yet naturalistic, situation for us to compare electronic social communication in adults with and without ASC.

We have observed a noticeable lack of social niceties and preamble in e-mail responses from autistic participants, yet an equally polite and strong adherence to formal address (e.g., Dear Dr....). We also noted considerable attention to detail, often demonstrated by autistic participants correcting the experimenter (e.g., grammatical errors or notifying us about broken URLs in our e-mail signatures), or correcting themselves if they found typographical errors in a previous e-mail (e.g., misspellings our names or locations). Additionally, we noted that autistic participants very often communicated precise, though socially unconventional, information about their estimated time of arrival (e.g., 14:08), or they described locations in ways (e.g., using map coordinates) that never occurred during electronic interactions with matched neurotypical controls. As a corollary to examination of e-mail communications with autistic participants, we also note similar observations while communicating with our university students with ASC. Compared to neurotypical students, they report experiencing considerable difficulties in writing socially relevant electronic communications to their peers and academic staff and often report misinterpreting messages or being misinterpreted by others, which can lead to breakdown of two-way communication. In addition, many autistic students are more unresponsive than non-autistic students, which they report is due to fatigue in filtering through ‘e-mail newsletters’ to identify important e-mail communications. Indeed, much of our time spent in supporting autistic students involves helping them to write and be more responsive to electronic communications and deal with negative consequences (e.g., anxiety) of actual or perceived ‘electronic faux pas’.

Our observations are broadly consistent with research on enhanced attention to detail [3] and time perception [4] in ASC. Equally, our observations follow evidence that social motivation may be atypical in some autistic individuals, for example showing reduced propensity to moderate their electronic behaviour to manage how they are perceived by the experimenter despite being able to do so [5]. Finally, we note that electronic social communication difficulties, particularly from our mentoring of autistic students, appear to arise from difficulties in understanding and appropriately responding to others’ mental states, in line with theory of mind-based explanations of ASC [6] or from having their atypical communication style misinterpreted by neurotypical people, in line with the double empathy theory of ASC [7].

In view of our observations, which are so far largely anecdotal, but nevertheless reflect theoretical and empirical work on ASC, we tentatively propose that atypical electronic social-communicative behaviour in autism is in line with many of the social-communicative features of ASC observed during social interactions in everyday life. We suggest that formal, rigorous investigation of electronic social communication in ASC is now required to ascertain if it is a broader characteristic of ASC. This could provide an important avenue for understanding autistic adults and call for improved efforts to help autistic people (and neurotypical e-mail readers) to improve communication over e-mail. We are submitting these observations as a *Letter to the Editor* in view of several potential issues with conducting an empirical study on the topic. For example, there are ethical constraints of analysing and publishing private messages with our participants and students. Obtaining ethical approval for an empirical study on electronic social communication in ASC would require informed consent about the research, which would disrupt the naturalistic communication with (autistic and non-autistic) participants because they would likely moderate their behaviour after becoming aware their e-mails were to be monitored and analysed. Other issues include difficulties in controlling for extraneous environmental factors, such as the amount of time taken to write the messages, devices used (i.e., phones versus laptops) and the surrounding environment, which may all affect the amount of attention and interest in composing effective electronic communication. Herein lies just some challenges for advancing research on this interesting topic, but it is hoped that this paper provides the impetus and potential avenues for investigating electronic communication in ASC.

We suggest three possible directions for future research on this topic. First, it should be possible to conduct carefully designed experimental studies in which participants *believe* they are composing real e-mails to others in order to assess autistic people’s electronic communication patterns, and potentially, neurotypical people’s (mis)understanding of these correspondences. Second, there are numerous other forms of electronic communication by autistic people in the public domain (e.g., online forum and social media posts), which could potentially be systematically analysed. Finally, and more straight forwardly, research could directly investigate autistic people’s self-reported difficulties (and anxiety) surrounding electronic communication in order to improve support, for example, for autistic university students.

We propose that this line of inquiry into electronic communication in autism could ultimately inform novel ways to help identify ASC (e.g., by measuring atypical electronic as well as face-to-face social communication) and support autistic people (e.g., by training professionals about potential e-mail miscommunication with autistic people). Overall, the topic warrants greater attention in future research and clinical and educational practice.

## Data Availability

N/A

## Abbreviation

ASC: Autism spectrum conditions
